# Optimizing Occupant Comfort in a Room Using the Predictive Control Model as a Thermal Control Strategy †

**DOI:** 10.3390/s24123857

**Published:** 2024-06-14

**Authors:** Mihaela-Gabriela Boicu, Grigore Stamatescu, Ioana Făgărăşan, Mihaela Vasluianu, Giorgian Neculoiu, Marius-Alexandru Dobrea

**Affiliations:** 1 Faculty of Hidrotechnics, Technical University of Civil Engineering Bucharest, 020396 Bucharest, Romania; giorgian.neculoiu@utcb.ro (G.N.); marius.dobrea@utcb.ro (M.-A.D.); 2 Faculty of Automatic Control and Computer Science, The National University of Science and Technology POLITEHNICA Bucharest, 060042 Bucharest, Romania; grigore.stamatescu@upb.ro (G.S.); ioana.fagarasan@upb.ro (I.F.)

**Keywords:** thermal comfort, predictive control model, fuzzy logic control

## Abstract

Thermal comfort strategies represent a very important aspect when it comes to achieving thermal comfort conditions. At the same time, recently, there has been a growing interest in user-centered building control concepts. Thus, this work focuses on developing a thermal control strategy that combines the restrictions related to achieving thermal comfort, expressed in terms of environmental parameters and specific factors of personal perception, with the objective of reducing energy consumption. This case study aims at implementing this strategy in a laboratory room located within the Technical University of Civil Engineering Bucharest. The strategy proposed by the authors is based on implementing a combination of a Model Predictive Control (MPC) model and a fuzzy system, which presents constraints related to the room occupancy level. Relevant observations regarding the parameterization of fuzzy systems are also highlighted.

## 1. Introduction

In recent decades, the focus in the field of smart buildings has increasingly shifted towards the use of modern ICT (Information and Communication Technology) to ensure a comfortable indoor environment for building occupants, with energy-efficient restrictions, in climate-neutral conditions [1,2,3]. In this context, the concept of occupant-centric thermal comfort has become a priority for engineers, designers and building managers. While the traditional objective of heating, cooling and ventilation systems has been to maintain technically acceptable thermal conditions, the current emphasis has shifted towards creating an environment that meets the individual needs and preferences of users [4,5].

This new perspective highlights a growing understanding of the fact that thermal comfort cannot be simplistically defined solely by technical parameters such as air temperature and humidity. It is also necessary to consider human and behavioral aspects. Different heating methods can significantly influence the personal perception of comfort, as certain methods can provide a more uniform and natural warmth, while others may create uneven temperature zones or sensations of discomfort [6,7,8]. Therefore, effective thermal comfort control is not only about establishing ideal environmental conditions, but also involves understanding the needs and preferences of occupants, adapting control systems accordingly [9,10].

In the age of digital and smart technology, the user-centric building control concept represents an emerging paradigm in the design and management of the built environment [11]. This approach aims to put the user in the center of the process of controlling and regulating the building’s indoor environment, in order to create personalized and comfortable experiences according to individual needs and preferences. In a constantly developing world, where people spend more time in indoor spaces, the importance of thermal comfort in buildings is becoming more obvious. Thermal comfort is thus a fundamental element for users’ quality of life and well-being, having a significant impact on their health, productivity and satisfaction [12].

It is important to note that this thermal comfort may vary depending on individual factors and the specific context of use of the building. For example, thermal comfort for people active in a gym may differ from thermal comfort for people who work in the office or for those who relax at home. Thus, assessing and ensuring thermal comfort in a building requires consideration of the needs and preferences of the occupants, as well as the specific characteristics of the building and the environment.

Fuzzy-based building control and the thermal index developed by Fanger represent an advanced and efficient approach for managing environmental conditions inside a building, with a focus on user comfort and well-being [13,14]. Fuzzy systems allow modeling and simulation of human reasoning in situations where there is uncertainty and ambiguity. In building control, these systems can be used to make smart and adaptive decisions regarding the management of environmental conditions. By dynamically adapting to environmental conditions, these systems can help increase energy efficiency and reduce environmental impact. This combined approach provides a robust and efficient way to manage thermal comfort in buildings, considering both technical and human aspects.

One of the constraints of minimizing electricity consumption relates to the occupancy level of a room, which is intermittent in most cases. Considering this aspect, it is recommended that the reference point for indoor temperature be variable so that comfort levels during room occupancy periods are not affected by these savings. Classical control strategies that involve only monitoring and ensuring system stability are no longer sufficient to obtain satisfactory conditions for thermal control expressed according to the aforementioned restrictions. Thus, the proposed objectives can be achieved by adding additional conditions in the defined control strategy to reduce the impact of disturbances in the system. These conditions relate to the use of the room occupancy schedule, but also to the consideration of factors related to the weather forecast [15,16].

The implementation of thermal control strategies for rooms involves the use of algorithms and techniques with which interior temperature can be regulated efficiently and precisely to ensure an optimal level of comfort for occupants and minimize energy consumption. These strategies may include the use of smart thermostats [17], temperature sensors and automated control systems. One of the advanced thermal control models is the predictive control model. This strategy is based on the use of a mathematical model of the heating process, which allows predicting its behavior in the future [18]. By considering external environmental conditions, the building occupancy schedule and other relevant variables, the predictive control algorithm can generate an optimal sequence of commands for the heating system to ensure optimal thermal comfort and high energy efficiency. The implementation of these advanced thermal control models can bring significant benefits in terms of occupant comfort and reduced energy costs in buildings.

The authors of the paper [19] focus on describing and analyzing the user-centered building control concept, an approach that puts the focus on user needs, preferences and behaviors in the building design and management process. Through advanced building automation and management technologies, such as intelligent lighting, heating and cooling, ventilation and safety control systems, user-centered building control aims to enable users to adapt the building’s indoor environment to their individual preferences and the specific needs for engaging in different activities.

## 2. Methods for Calculating the Comfort Index

This chapter presents a comparative study between the two most used expression indices of thermal comfort, considering the main factors that intervene in their definition. This part describes how both indices are calculated and how they are influenced by component parameters such as air temperature, relative humidity and other relevant variables that are analyzed and compared. Through this detailed analysis, we want to provide a comprehensive perspective on the effectiveness and applicability of each index in the context of thermal comfort assessment, in order to facilitate an informed choice and a deeper understanding of this concept.

Also, in this chapter, information regarding the collection and processing of data necessary for the developed study is presented, as well as details regarding the sensors used for measurements and other aspects related to the information collection process.

### 2.1. Thermal Comfort Indices: FANGER and Van Zuilen Indices

As specified, thermal comfort is defined by all of the microclimate conditions in a room that determine a pleasant ambience, in which the occupants are satisfied with the temperature of the environment. This is a subjective state, which implies that they consider the surrounding temperature to be pleasant or comfortable enough to carry out their activities. Thermal comfort is influenced by several factors, the most important being indoor air temperature, indoor air humidity, average radiation temperature, air velocity, clothing level of the occupant and intensity of physical activity.

One of the most relevant factors determining thermal comfort is indoor air temperature. At the same time, this parameter is of particular importance regarding the energy of the entire building, given that the indoor air temperature defines the energy consumption regarding the heating and cooling of rooms. Thus, the indoor air temperature corresponding to an occupant who is dressed appropriately for the season and not physically active is considered to be 20–22 °C in winter and 22–26 °C in summer.

Another essential factor in determining the level of thermal comfort in air-conditioned rooms is the level of air humidity. Generally, if the relative humidity of indoor air exceeds 70%, especially in the cold period of the year, this favors the formation of condensation on the inner surface of the walls. Therefore, the limits of the permissible level specific to the relative humidity in rooms are situated in the range of 35–70%.

The average radiation temperature is calculated by considering the average temperature of the respective surfaces of walls, windows, the ceiling, the floor and heating bodies in the room. The human body’s heat exchange occurs based on these temperatures. In conclusion, the average radiation temperature should be as close as possible to the indoor air temperature. This is achieved by correctly sizing heating bodies, which require a high radiation surface and high surface temperature, as well as by proper insulation of external walls and windows. 

Thermal comfort in ventilated rooms is influenced by the speed of air movement. If the temperature of the air in motion is lower than the ambient temperature, a feeling of discomfort for the occupants occurs. Therefore, the air velocity should range from 0.15 m/s to 0.25 m/s at typical indoor air temperatures of 20 °C to 22 °C. Higher air velocity values may be accepted when an occupant is active and suitably dressed.

The last two factors specified, namely the level of clothing and the physical activity intensity, are subjective factors, representative of the occupants of the room. Clothing has a special influence on the feeling of comfort through the thermal insulation offered by the outfit. The intensity of physical activity determines the amount of heat released by the occupant into the environment. In order to obtain the feeling of comfort, there must be a dependence between the level of activity and the temperature of the room.

A thermal comfort index is used to quantify the degree of thermal comfort in a given environment. Therefore, in order to assess the degree of thermal comfort of a room, indicators are used that sum up the effects of the factors described above. According to studies performed by Fanger [20], the thermal comfort assessment indices in environments with normal parameters combine the effects of at least two factors. 

Although there are multiple such indicators used to express thermal comfort, this study will point out the differences between two of them: the thermal comfort index developed by Van Zuilen and the model-specific indices developed by Fanger.

#### 2.1.1. Van Zuilen Model

**Figure 1:**
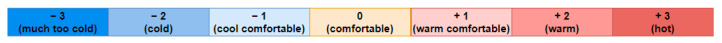
Van Zuilen index scale.

The model-specific thermal comfort index B developed by researcher Gerard Van Zuilen is based on two factors: indoor air temperature and average radiant temperature. The feeling of thermal comfort is assessed in this case by means of a subjective seven-level comfort scale, as shown in Figure 1.

Thus, it can be seen that the occupant is feeling thermal comfort when the values are between −1 and 1. The recommended values characteristic of the comfort index B depending on the type of activity and the purpose of the building are specified in Table 1. 

The feeling of thermal comfort is defined by the comfort index B, which is calculated as follows:

B_z_ = C_z_ + 0.25 × (t_i_ + t_mr_) + 0.1x_i_ − 0.1 × (37.8 − t_i_) √(v_i_) (1)

where

C_z_—constant considered to have the value −9.2 in the cold period and −10.6 in the warm period;
t_i_—indoor temperature [°C];
t_mr_—average radiation temperature of the room [°C];
x_i_—humidity of indoor air [gr/kg dry air];
v_i_—speed of indoor air currents [m/s].

A paper that uses this comfort index to express the user’s level of satisfaction in terms of thermal comfort is [21], in which the necessary environmental data for the study, namely the air temperature, relative humidity, user activity, clothing level and skin temperature, were collected through Fitbit bracelets. The article presents the stages of developing a fuzzy system with the specified parameters as inputs, the system’s output representing the user’s thermal comfort level, expressed according to Van Zuilen’s scale. The conclusion of this study emphasizes that the implementation of this system yielded satisfactory and relevant results. 

#### 2.1.2. Fanger’s Model

One of the most common and used models in studies is the model developed by Fanger. This model, developed by O. Fanger in the 1970s, is the foundation of several applicable standards, the best-known being ISO 7730 [22,23,24,25]. Based on this model, the following two specific indicators are defined: PMV—Predictive Mean Vote and PPD—Predictive Percentage of Dissatisfied. The PMV comfort index can be calculated for different combinations of energy metabolism, clothing level, indoor air temperature, average radiation temperature, air velocity and humidity according to the following formula:

PMV = (0.33 × e^−0.036M^ + 0.2028) × {(M − W) − 3.05 × 10^−3^ × [5733 − 6.99 × (n − w) − p_a_] − 0.42 × [(M − W) − 58.15] − 1.7 × 10^−5^ × M × (5867 − p_a_) − 0.0014∙M∙(34 − t_a_) − 3.96 × 10^−8^ × f_cl_ × [(t_cl_ + 273)^4^ − (t_r_ + 273)^4^] − f_c_ × h_c_ × (t_cl_ − t_a_)} (2)

where each factor represents the following:

M—energy metabolism [W/m^2^]
W—external activity [W/m^2^]
t_a_—indoor air temperature [°C]
t_r_—average radiation temperature [°C]
v_ar_—relative velocity of air in relation to the human body [m/s]
p_a_—partial water vapor pressure [Pa]
l_cl_—thermal resistance of clothing level [ m^2^ K/W]
f_cl_—the ratio between the clothed body surface area and the exposed body surface area
h_c_—convection heat transfer coefficient [W/m^2^ °C]
t_cl_—surface temperature of clothing [°C]

The following parameters are defined as follows:

t_cl_ = 35.7 − 0.028 × (M − W) − I_cl_ {3.96 × 10^−8^ × f_cl_ × [(t_cl_ + 273)^4^ − (t_r_ + 273)^4^] + f_cl_ + h_c_ × (t_cl_ − t_a_) (3)

h_c_ = 2.38 × (t_cl_ − t_a_)^0.25^ for 2.38 (t_cl_ − t_a_)^0.25^ < 12.1 × √v_ar_ or h_c_ = 12.1 × √(v_ar_) for 2.38 × (t_cl_ − t_a_)^0.25^ < 12.1 × √v_ar_ (4)

f_cl_ = 1.00 + 1.290 × I_cl_ for I_cl_ ≤ 0.078 m^2^ × C/W or f_cl_ = 1.05 + 0.645 × I_cl_ for I_cl_ > 0.078 m^2^ × C/W (5)

The PMV index represents the average opinion of a group of occupants who vote their perception of the sensation of thermal comfort expressed according to a scale with seven levels, defined in the range −3 to +3, where the negative range is specific to the sensation of cold, the positive range is specific to the sensation of heat and the neutral sensation of comfort is specific to the value 0, according to the American standard ASHRAE 55 [26]. According to Fanger’s theory, a room exhibits the characteristic of thermal comfort when the values of the PMV index are in the range of −0.5 and 0.5. The determined PMV will be valid for steady-state conditions but may be estimated with a good approximation when one or more variables vary slightly, provided that their averages are considered time-weighted over the preceding hour.

We can see that both models present advantages and disadvantages, and they can be used together or separately. The main difference between the model defined by Fanger and the model defined by Van Zuilen is that the latter model is a simplified one, suitable for spaces with few heat sources or low variability in temperature and air velocity, while the Fanger model is used more in complex environments, such as large rooms or industrial spaces.

Another difference occurs in the formula for calculating the PMV index, where, compared to the one for calculating the B index, two very important factors appear: energy metabolism and thermal insulation from the clothing level. Considering this aspect, in this paper the representation of the comfort level obtained was chosen based on the model defined by Fanger that includes the two parameters specific to this model: the occupant’s activity level and clothing level.

### 2.2. Data Used

The study presented in this paper is based on a dataset that was collected by the authors using a Sencor SWS 12500 WiFi model weather station, this model being produced by the SENCOR manufacturer with the address in Ricany, Czech Republic. This station is compatible with smart devices and provides access via the internet to history, data and global public weather platforms and present built-in sensors that measure wind speed and direction, precipitation, indoor and outdoor temperature, indoor and outdoor humidity, light intensity and UV radiation.

With the help of this device, data relating to internal and external environmental factors were retrieved and stored, from which parameters of interest for this study were selected, namely indoor temperature and humidity, and outdoor temperature and humidity, respectively. The technical description of the device used is given in Table 2.

**Figure 2:**
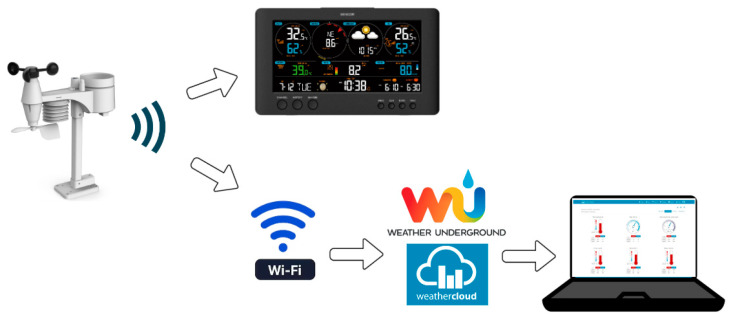
Data transmission from the weather station to the user interface.

The weather station used has an LCD screen that displays in real time the data taken from it, which presents a user-friendly interface. Figure 2 shows how to transmit and retrieve the datasets recorded by the weather station.

## 3. Methodology

This chapter provides information on the two main components of the proposed system. The first component consists of a fuzzy system that presents as inputs both indoor environmental parameters and parameters representative of the personal perception of the occupants. In the first part of this chapter, conclusions regarding the parameterization of this system are presented, highlighting the essential aspects of its configuration and optimization to ensure efficient and precise operation.

In the second part of this chapter, the concept of the predictive control model, which represents the second component of the proposed system, is detailed. The principles and methods associated with the predictive control model are analyzed in depth, highlighting its advantages and applications in the context of thermal control strategies. This approach is then integrated with the fuzzy system to form the proposed HVAC system control solution. 

### 3.1. Fuzzy System Parameterization

In this subchapter, we aim to highlight the differences that were encountered from the parameterization of the developed fuzzy system and to conclude with the choice of the right version for our study implementation. The fuzzy system that results in the level of thermal comfort expressed according to the scale defined by Fanger follows the structure of article [22], where inputs are represented by two types of factors, namely environmental factors (indoor temperature and humidity) and factors related to personal perception (skin temperature, level of clothing and level of activity performed). In previous studies [21,22], the developed fuzzy system presents defined inputs using Gaussian membership functions (GFMFs) and a triangular membership function, specific to the output of the system, namely the level of comfort obtained. The fuzzy system is the Mamdani type, developed using the Matlab/Simulink toolbox. In this study we used Matlab version R2020a (MATLAB 9.8). The defuzzification method implemented is the centroid method. Also, the system presents a rigorously defined rule base, the results of the simulation obtained being satisfactory. 

However, this chapter aims to highlight the variations in the response obtained after parameterizing the original defined system, in order to identify the most optimal variant of its implementation. Below, the ranges defined for each parameter considered are described, as well as the optimal value for achieving the state of thermal comfort.

Environmental factors refer to the parameters measured inside the studied room. The temperature in this context represents an input to the fuzzy system and is defined in the range 15 °C–33 °C, where the optimal range considered necessary in a classroom has been chosen as 20 °C–24 °C. In correlation with the temperature value, the second indoor parameter, relative humidity, has been defined so that it can take values between 0% and 100%. The considered comfort range for relative humidity is between 40% and 60%.

The skin temperature is defined in the range 34 °C–40 °C, considering the following cases: The normal temperature of a person is situated between 36 °C and 37 °C. If the temperature falls below 35 °C, then the person presents a state of hypothermia, and if the temperature rises above 38 °C it is considered that the person is in a febrile state. Representative labels have been considered to define the following two parameters. The level of clothing includes four possible cases, which are defined in Table 3.

Defined in a similar way, the labels specific to the level of activity carried out in the classroom are the following: sitting = 1, standing = 2, walking = 3, light activity = 4.

#### 3.1.1. Version 1

**Figure 3:**
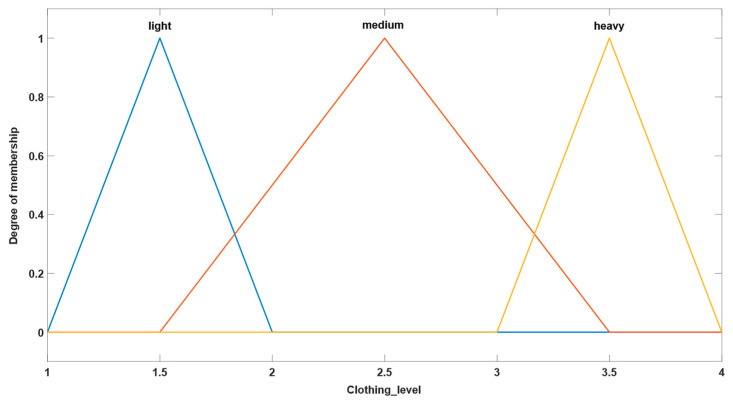
Example of triangular-type input.

**Figure 4:**
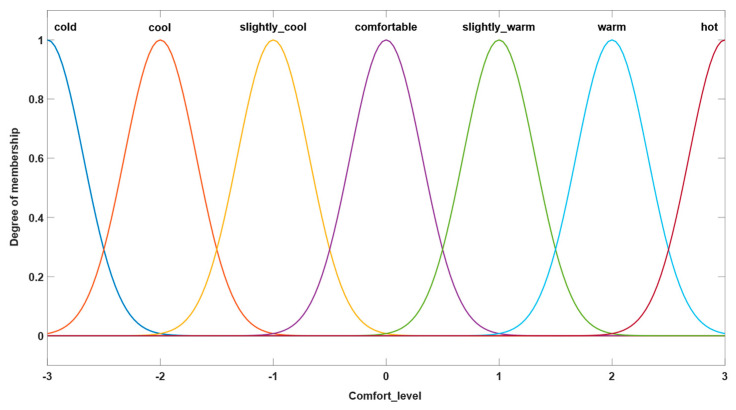
Example of Gaussian-type output.

In the first parametrization version of the fuzzy system, a combination of defined inputs of triangular type and defined outputs of Gaussian type were utilized. The types of membership functions used in this parameterization version can be seen in Figure 3 and Figure 4, which show their characteristic graphs. The rules database of the fuzzy system comprises the same rules defined as in the original system, the purpose of this study being to focus on observing the differences that occur because of the changes in membership functions representative of system inputs and outputs.

By exploring these differences, we aim to understand how the changes to membership functions can influence the behavior and performance of the fuzzy system in various scenarios and operating conditions.

#### 3.1.2. Version 2

**Figure 5:**
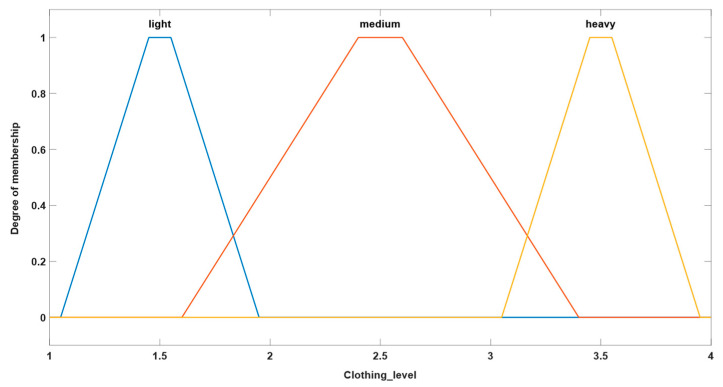
Example of trapezoidal-type input.

**Figure 6:**
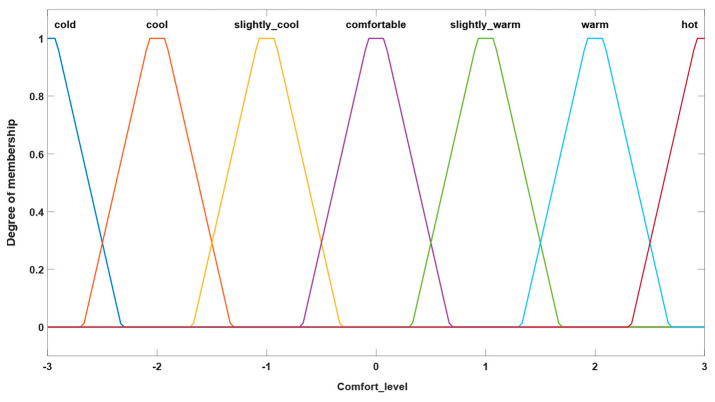
Example of trapezoidal output.

The second version for parameterization of the studied fuzzy system involves defining both inputs and outputs by means of trapezoidal membership functions. The rule base of this system is the same as that mentioned in the previous examples. Thus, Figure 5 and Figure 6 illustrate the types of inputs and outputs chosen for this system.

We mention that the ranges for defining both input and output sizes have been established in accordance with the thresholds defined in the introduction of this chapter. This approach ensures consistency between the values associated with the inputs and output of the system and the decision criteria defined through the initial rules, contributing in this way to an efficient and consistent implementation of the fuzzy system in the analysis carried out. Alignment of definition ranges with predetermined thresholds facilitates interpretation and adjustment of system behavior in accordance with proposed requirements and objectives.

### 3.2. Results Obtained

**Figure 7:**
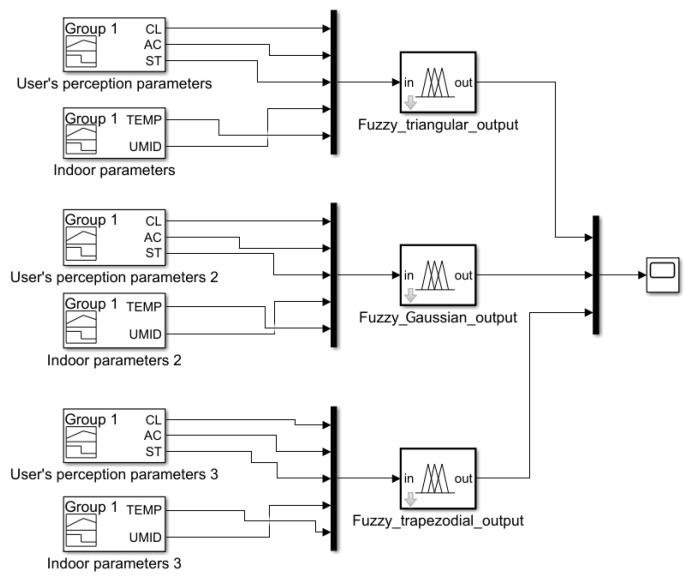
Scheme of implementation for the response of the three fuzzy system versions.

The main scope of the fuzzy system’s initially proposed parametrization involves highlighting the differences between the three proposed versions and concluding which of them presents the best performance in terms of the occupant’s comfort level. In order to compare these versions of the fuzzy system, a simulation was performed using the MATLAB/Simulink development environment, according to the scheme shown in Figure 7 below.

**Figure 8:**
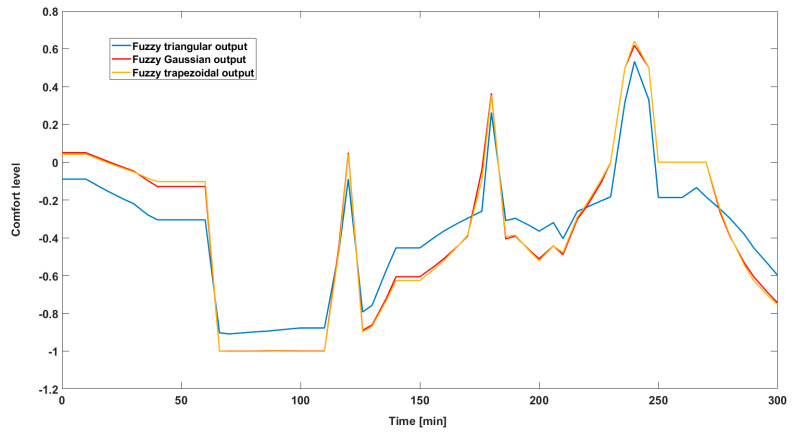
Comparison of the three types of membership functions characteristic of the system output.

Figure 8 presents the results obtained from simulating the three versions of the fuzzy system in terms of its output (comfort level obtained). We can highlight the fact that the results obtained relate strictly to the output size and how it was defined. We mention that this simulation was carried out relying on a time interval of 4 h, characteristic of the scenario from the dataset described in Section 4.2.

From the graph obtained, we can first see a significant variation between the response of the system that has defined the output shape as triangular (blue curve), namely the original system, and the other two curves. Taking this into consideration, and the fact that the two almost identical curves are representative of the outputs defined by Gaussian and trapezoidal curves, there is a direct link to the variation specified above.

Also, sudden oscillations produced over short periods of time can negatively affect the rest of the process. These are mainly due to variations specific to the input parameters, which directly affect the response of the system. According to the dataset used in this example, we can deduce that these variations are due to the level of activity carried out by the occupant, as this is the only parameter that shows these sudden changes.

Another aspect regarding the resulting graph relates to the positioning of the curve obtained for the defined triangular membership functions (blue color) compared to the graph resulting from the implementation of Gaussian membership functions (red color). Because of this aspect, we can see that during the first hour, the representative graph of the triangular curve is positioned below the representative graph of the Gaussian curve. At the comfort threshold obtained for the value −1, however, a change in the positioning of the two types of curves mentioned is observed, in which case the triangular curve is positioned above the Gaussian curve. This aspect shows the differences obtained in terms of parameterization of membership functions specific to fuzzy system output.

We can also see that, if the graph presented had illustrated the dependence of input variables, it would have been possible to identify the correlation between the curve represented by the blue color, which denotes the inputs determined based on triangular membership functions, and the curve represented by the yellow color, which represents the inputs defined as trapezoidal-type within the system. This observation highlights the differences between how input variables are modeled and how these patterns influence system behavior. 

### 3.3. Model Predictive Control

**Figure 9:**
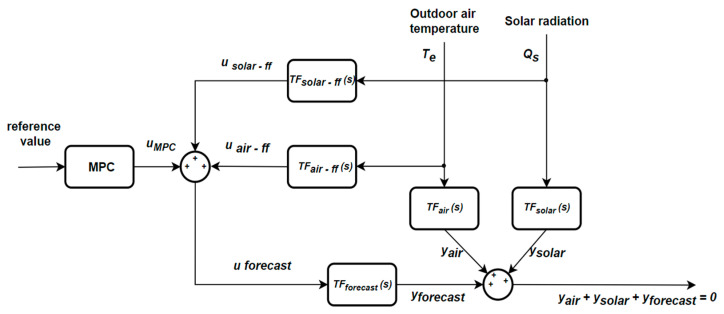
General block diagram of a prediction system.

One of the most important aspects related to an indoor environment from a thermal point of view relates to the steps that must be considered to obtain its thermal load, which can be approached as a control problem that is achieved in two stages. The first stage involves calculating the thermal load of the room with the restriction of eliminating the disturbing effects of the outdoor temperature and solar radiation. To achieve this result, a feedforward control strategy can be implemented to compensate for the effects produced by the mentioned parameters, which will obtain the amount of heat necessary to maintain the optimal comfort level. In the second stage, the calculation of the thermal load is carried out so that it follows as closely as possible the reference value for the interior temperature. One possibility of implementation is represented by the classic version of control that uses PID controllers. However, to obtain more accurate results, it is recommended to consider both weather forecast and room occupancy schedule data, so that a predictive control strategy is used, as is represented in Figure 9.

The abbreviations used in the figure above are presented below:

MPC—Model Predictive Control
u—input parameter
u_ff_—input parameter in feedforward
TF (s)—transfer function
T_e_—outdoor air temperature
Q_s_—solar radiation
y—output parameter

As is mentioned in [27], within the control strategy, correcting disturbances caused by weather conditions affecting the building can be achieved by using a feedforward control strategy. Thus, the effect of disturbances is eliminated before they have visible effects on the system. By employing the transfer function that expresses the influence of external temperature on the system output (the indoor temperature), corresponding to TF_air_(s) from Figure 9 and the values of the disturbance signal represented by the outdoor air temperature, T_e_, the value of the output variable on the system in the form of a disturbance to the influence of outdoor air temperature y_air_ can be determined according to the following equation:

y_air_ = TF_air_(s)·T_e_ (6)

Therefore, to eliminate the effect of these disturbances, it is necessary for the output resulting from the disturbing effect of the external temperature to be compensated for by the output resulting from the effect imposed by the system command, y_forecast_:

y_air_ + y_forecast_ = 0 (7)

By applying the inverse method for solving control problems, the value of the input variable u_forecast_ can be determined using the transfer function TF_forecast_(s) and the desired evolution of the command y_forecast_. Thus, Equation (8) is obtained.

u_forecast_ = TF_forecast_(s)^−1^·y_forecast_ (8)

Thus, it can be concluded that the feedforward transfer function for compensating for the disturbances caused by the external temperature has the following form:

TF_air-ff_(s) = TF_forecast_(s)^−1^·TF_air_(s) (9)

In addition to the disturbing factor introduced by the external temperature, another disturbing factor is the incident solar radiation on the building envelope, Q_s_. Proceeding similarly to the compensation for the disturbance caused by the external temperature, the feedforward transfer function for compensating for the disturbances introduced by solar radiation takes the following form:

TF_solar-ff_(s) = TF_forecast_(s)^−1^·TF_solar_(s) (10)

Considering that the system’s disturbing factors are those previously mentioned, compensating for weather conditions in the system can be achieved by applying the total compensation command, represented as follows:

u_forecast_ = TF_forecast_(s)^−1^·TF_air_(s)·T_e_ + TF_forecast_(s) − 1TF_solar_(s)·Q_s_ (11)

When it comes to intermittently heated buildings, the reference temperature shows a step variation, while the temperature felt in the room shows a much slower transition. The purpose of the implemented controller is to make the transition between the two temperatures, calculating the heat flow necessary to obtain thermal comfort through minimum energy consumption.

**Figure 10:**
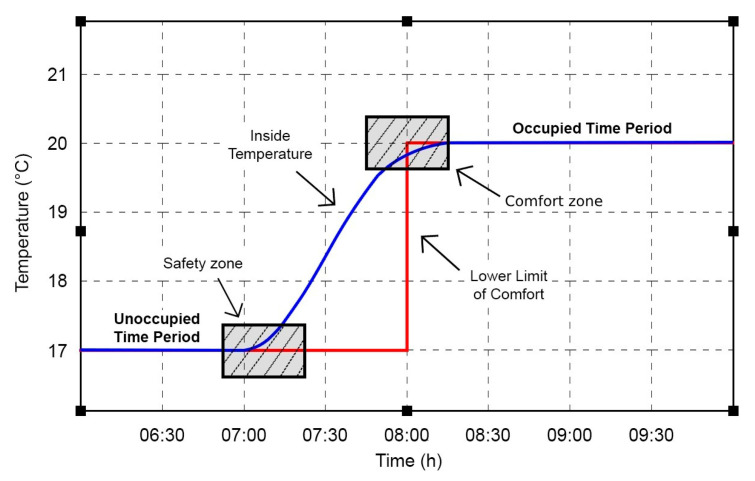
Graphical representation of the comfort zone and the safety zone.

The analyzed problem is reduced to determining the right moment to turn on the heating, so that the comfort level of the occupants is reached at the beginning of the occupancy period of the room, while having minimum energy consumption. The ideal start-up time varies and depends on several factors such as weather conditions, outdoor and indoor temperature, thermal characteristics of the building and maximum power of the heating system. Thermal comfort is defined in the form of a temperature range, which presents a lower and an upper limit, in which the main parameter, indoor temperature, should be situated. This interval varies depending on the period of occupancy of the room. When the room is occupied, we take into consideration the comfort zone, and when the room is empty, the safety zone, according to Figure 10. From the perspective of fulfilling the restriction regarding the minimization of energy consumption, it can be observed, however, that there is a need to maintain the temperature range in the comfort zone.

In order to exclude the case that at the beginning of the occupancy period the indoor air temperature could fall below the comfort zone, the heating process should start in advance for a certain period of time. This aspect also considers the notions related to the dynamics of the building, which have high inertia. It can be concluded in this regard that when the indoor temperature graph intersects with the control area of the optimal starting point, according to the figure above, the conditions for the optimal start of heating are met. The maximum allowable variation in this control area, which also depends on when the occupancy status of the room changes, is within 1 °C over a period of 30 min. This variation relates to the lower limit of comfort and is situated in the range of ±0.5 °C of its value.

One of the most advanced directions of automatic temperature control is Model Predictive Control (MPC). It involves a class of control algorithms that optimize future process behavior by calculating a specific command sequence. MPC is an advanced control method used to dynamically adjust systems in real time and uses a mathematical model of the controlled system to predict its behavior in the future based on a series of possible control inputs. 

## 4. Case Study

The data needed for this study were collected over a period of several months, within the Technical University of Civil Engineering Bucharest, specifically the Faculty of Hydrotechnics, which is geographically positioned in the southeastern area of Muntenia, Romania. Data on indoor environmental parameters were collected from a classroom located on the second floor of the building.

**Figure 11:**
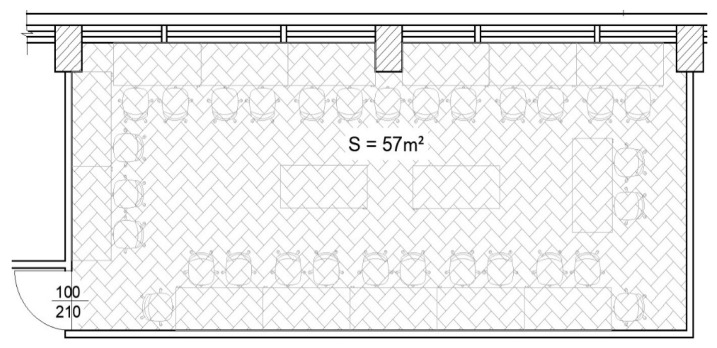
The plan of the classroom.

Being a room designed for laboratory activities, it has a capacity of approximately 30 students, distributed at 14 workstations that are located over the entire surface of the room, the total surface being 57 m^2^. Both the plan and the location of the room where the experiment took place can be seen in Figure 11. In this context, the sensors were placed in the center of the room, at the height of the worktables, to measure the average conditions in the room.

**Figure 12:**
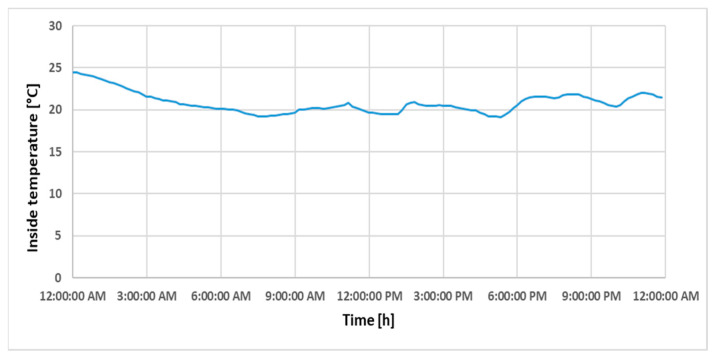
Variation in indoor temperature during a day.

**Figure 13:**
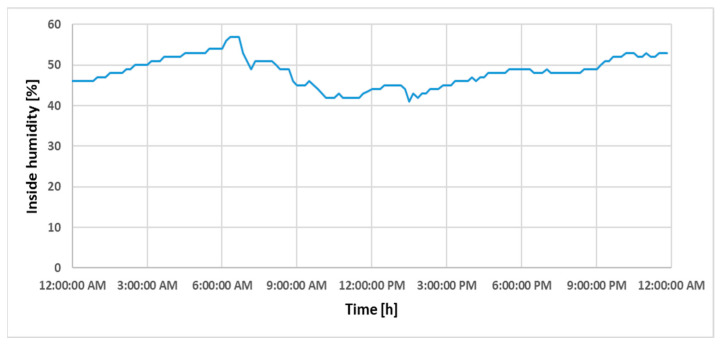
Variation in indoor humidity during a day.

In order to validate the strategy and interpret the simulations, we suggested choosing data recorded during a weekday, so that there was activity in the classroom. Thus, the analyzed dataset is specific to Tuesday, 14 November 2023, this being an autumn day with positive temperature records throughout the entire interval. In Figure 12 and Figure 13, the variations in measured indoor temperature and humidity during the time interval of interest of this study can be seen, with the time interval starting at 7:00 a.m. and ending at 08:00 p.m.

### 4.1. Test Scenario

From the point of view of parameters representative of users’ personal perception (skin temperature, activity level and clothing level), test scenarios were used for the developed control model, and in the future we will implement the retrieval of specified parameters using wearable devices. We mention that the values considered for testing represent an average of the majority vote specific to skin temperature parameters, activity level and clothing level.

Although the necessary data were collected throughout the day, we selected from the data only the interval of interest for our study, namely the interval in which teaching activities took place in the classroom. We considered the period for testing the model as between 07:00 a.m. and 09:00 p.m., a period correlated with the occupancy of the room. During this period, both practical and maintenance activities were carried out. Table 4 shows the experimental data collected over a period of 90 min.

The first laboratory in this room takes place between 08:00 a.m. and 09:50 a.m., being a theoretical laboratory where the main activity carried out by students is individual work. Thus, a change in the activity level is made only in the interval intended for breaks, namely 8:50 a.m. to 9:50 a.m., when from the specific position of the learning activity—label 1, this representing the stationary position at the office—changes to label 3 occur, which is equivalent to a level of activity corresponding to walking.

The second laboratory takes place between 10:00 a.m. and 11:50 a.m., being a laboratory where the main activity is individual work carried out by the students at the workstations. In the continuation of this laboratory, there is a time period of an hour in which the room is empty, and then the third laboratory takes place between 01:00 p.m. and 02:50 p.m. The activity specific to this interval is mainly working individually, with moments in which the students periodically walk to the blackboard to solve exercises. In these moments, the level of activity also changes according to the action carried out. The last laboratory of this day takes place between 06:00 p.m. and 07:50 p.m., an interval in which the main activity is individual work. Given that the maximum outdoor temperature reached 17 °C, but it was also an autumn day with a very low minimum temperature, in this test scenario an average clothing level (equivalent to a blouse/shirt) specific to label 3 defined in the fuzzy system was taken into consideration. We also specify that we considered that all participants had an optimal state of health and implicitly an optimal body temperature. According to this information, we considered it appropriate for this parameter to have a value of 36 °C.

### 4.2. System Architecture

For the aforementioned room, the mathematical model was made based on the specifications in [27], which describes a model that is used within the predictive control algorithm. The article studies the experimental identification of the dynamic heat transfer model in a building, which is essential for optimal temperature control and energy performance evaluation. Using heat equations in the time domain and matrix representations for multiple input and output systems, the state-space and transfer function representations are obtained. Additionally, the model parameters are determined. As a detailed example, the study monitors a series of internal and external parameters of a standard-sized house. By analyzing these predictions, the MPC determines the optimal sequence of control inputs to meet certain goals, such as minimizing error or optimizing the cost function [27,28,29]. MPC is used in a variety of fields, including in the chemical, automotive and robotics industries, due to its ability to manage complex and variable systems. 

Disturbance compensation and temperature stabilization are no longer satisfactory solutions to accomplish the current requirements imposed for thermal control of buildings. In order to solve the problems specific to control strategies and to achieve the main objectives proposed in this paper, namely ensuring thermal comfort with the constraint of minimizing energy consumption, this paper proposes the implementation of a thermal control strategy in a feedback loop that uses both the most important environmental variables and the dynamics of the system (history). The variables that are considered in the implementation are e_t_ (measured temperature), t_outside_ (outdoor temperature), t_predicted_ (weather forecast), history (occupancy schedule) and x (other types of information), according to the equation below.

t_air_ = f_MPC_ (e_t_, t_outside_, t_predicted_, x, history) (12)

**Figure 14:**
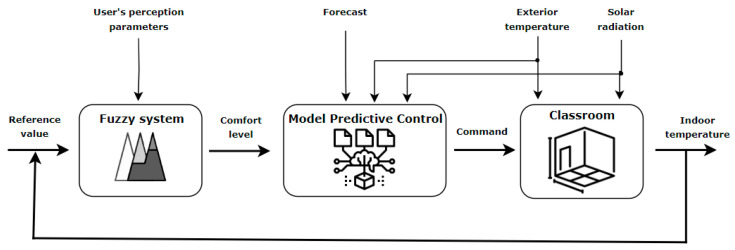
Architecture of the proposed system.

In this study, variable x is represented by the information obtained at the output of the fuzzy system, namely the comfort level of the occupants. The system also considers the room occupancy schedule according to the test scenario described above. The architecture of the proposed system is the one represented in Figure 14, which highlights the concept of implementing the fuzzy model predictive control (FMPC) algorithm. FMPC is an extension of MPC that integrates fuzzy logic concepts to deal with uncertainties and variability in the system model or control environment. Instead of relying strictly on precise mathematical models, FMPC uses fuzzy language rules to describe system behavior and make control decisions. These fuzzy rules are defined by experts or drawn from experimental data, allowing the FMPC to adapt to changes in the control environment and manage uncertain or imprecise situations. FMPC is used in areas where mathematical modeling of the system is difficult or uncertain, such as temperature control in buildings or robotic control in unstable environments.

Control strategies based on both MPC and FMPC have been successfully applied in a variety of fields, such as industrial automation, chemical process control, autonomous vehicles and robots. These techniques have demonstrated outstanding capabilities in improving the efficiency, safety and performance of controlled systems, contributing to the continuous advancement of technology [27,28,29].

**Figure 15:**
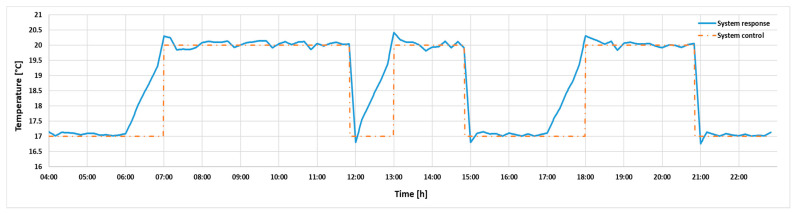
Results achieved.

Starting from the system architecture proposed above, for the dataset presented in this paper, the results for the system are shown in Figure 15, where the orange graph represents the system command and the blue graph represents the command sent to the heating, ventilation and air conditioning system implemented with MPC, expressed according to the occupancy level of the room.

As seen in the figure above, the system response corresponds to the requirement imposed for both the comfort zone during the occupied period and for the safety zone during the unoccupied period, the requirement being to ensure the maximum level of comfort with the minimum level of energy consumption being met.

## 5. Conclusions

In the first part, this paper presents the results from the parameterization of the membership functions specific to the inputs and outputs of the fuzzy system developed to achieve thermal comfort in correlation with the preferences of room occupants. Adjusting these parameters can significantly influence how the system responds to various input-specific conditions. Proper parameterization can enhance the accuracy, stability and adaptability of the fuzzy system. Additionally, this article presents the differences and dependencies that arise from the parameterization of these membership functions across three studied cases involving inputs and outputs defined by different curves. From this analysis, an initial conclusion is that for our study, the optimal fuzzy system was achieved with inputs defined by triangular or trapezoidal curves and outputs defined by Gaussian or trapezoidal curves, the differences between these two options being minimal.

The aim of this article is the implementation of a user-centered thermal control strategy applied to a classroom at the Technical University of Civil Engineering Bucharest. This study is based on indoor and outdoor environmental data collected using sensors integrated into the Sencor SWS 12500 WiFi station. Additionally, the study presents a testing scenario for the proposed model, which considers the occupancy level of a laboratory room. However, the model also shows applicability and feasibility in other scenarios or situations. Following the implementation of the proposed control strategy, results were obtained that comply with the imposed restriction of minimal energy consumption while ensuring optimal comfort. This is evidenced by the fact that at the beginning of each occupancy period, the time interval during which the indoor temperature exceeds the setpoint value is short and within the maximum comfort zone limit. Regarding the condition of maintaining occupant comfort, the results validated the developed control strategy, as during the occupancy period, the indoor temperature remained within the comfort zone specified in this study.

Thus, this paper presents the results of a thermal control strategy developed based on fuzzy logic and an MPC algorithm. The proposed solution fulfills the conditions regarding energy consumption minimization by maintaining room temperature within desired parameters, depending on the room’s occupancy period, and activating the heating–cooling system only in certain moments, without exceeding the maximum value. Additionally, according to Figure 15, it can be observed that the heating system starts earlier, ensuring that by the time activities start in the laboratory room, the temperature will be situated in the comfort range of the occupants. Consequently, the main objectives outlined in this paper have been achieved, yielding satisfactory results. 

## Figures and Tables

**Table 1:** Comfort index B values recommended for certain situations.

Room Purpose	Level of Activity	Heat Released by the Occupant [kcal/h]	Recommended Comfort Index B Value
Production halls/commercial halls/shops (t_i_ = 18 °C)	Light physical work	Under 200	−1.5 < B < −0.5
Administrative buildings/offices (t_i_ = 18–20 °C)	Computer work/reading/light activity	Under 155	−1.5 < B < −0.5
Houses/schools (t_i_ = 20 °C)	Human at rest/reading/light activity	100	−0.5 < B < −0.5

**Table 2:** Features of sensors built into the Sencor SWS 12500 WiFi station.

Feature	Range	Precision
Indoor temperature measurement	−5 to +50 °C	±1.5 °C
Outdoor temperature measurement	−40 to +60 °C	±1.5 °C
Indoor humidity measurement	1% to 90%	±5%
Outdoor humidity measurement	1% to 99%	±5 %
Sensor transmission distance	Up to 150 m	-
Transmission frequency	868 Mhz	-

**Table 3:** Definition of clothing level-specific labels.

No Label	Name	Clothing
1	Very light	Summer clothing
2	Light	T-shirt
3	Medium	Blouse, shirt
4	Heavy	Winter clothing—sweater

**Table 4:** Experimental data.

Date (Europe/Bucharest)	Day	Inside Temperature (°C)	Inside Humidity (%)	Skin Temperature	Activity Level	Clothing Level
8:00:00 a.m.	14 November	19.3	51	36	1	3
8:10:00 a.m.	14 November	19.3	50	36	1	3
8:20:00 a.m.	14 November	19.4	49	36	1	3
8:30:00 a.m.	14 November	19.5	49	36	1	3
8:40:00 a.m.	14 November	19.5	49	36	1	3
8:50:00 a.m.	14 November	19.6	46	36	3	3
9:00:00 a.m.	14 November	19.7	45	36	1	3
9:10:00 a.m.	14 November	20	45	36	1	3
9:20:00 a.m.	14 November	20	45	36	1	3
9:30:00 a.m.	14 November	20.1	46	36	1	3

## Data Availability

Data are contained within the article.
